# A new drug combination significantly reduces kidney tumor progression in kidney mouse model

**DOI:** 10.18632/oncotarget.26004

**Published:** 2018-08-31

**Authors:** Sitai Liang, Tiffanie Cuellar, Maciej Nowacki, Bijaya K. Nayak, Lily Dong, Boajie Li, Kumar Sharma, Samy L. Habib

**Affiliations:** ^1^ Department of Cell Systems & Anatomy, University of Texas Health Science Center at San Antonio, Bio-X Institutes, San Antonio, TX, USA; ^2^ Department of Medicine, University of Texas Health Science Center at San Antonio, Bio-X Institutes, San Antonio, TX, USA; ^3^ Shanghai Jiao Tong University, Shanghai, China; ^4^ South Texas Veterans Health Care System, San Antonio, TX, USA

**Keywords:** kidney tumor, rapamycin, AICAR, HIF2α, TSC2

## Abstract

Tuberous sclerosis complex (TSC) disease is associated with tumors in many organs, particularly angiomyolipoma (AML) in the kidneys. Loss or inactivation of *TSC1/2* results in high levels of HIF-α activity and VEGF expression. mTOR inhibitor (rapamycin) and the AMPK activator 5-aminoimidazole-4-carboxamide (AICA)-riboside (AICAR) are currently used separately to treat cancer patients. Here, we investigated the effect of a novel combination of rapamycin and AICAR on tumor progression. Our data show that treatment of AML human cells with drug combinations resulted in 5-7-fold increase in cell apoptosis compared to each drug alone. In addition, drug combinations resulted in 4-5-fold decrease in cell proliferation compared to each drug alone. We found that drug combinations abolished Akt and HIF activity in AML cells. The drug combinations resulted in decrease in cell invasion and cell immigration by 70% and 84%, respectively in AML cells. The combined drugs also significantly decreased the VEGF expression compare to each drug alone in AML cells. Drug combinations effectively abolished binding of HIF-2α to the putative *Akt* site in the nuclear extracts isolated from AML cells. Treatment TSC mice with drug combinations resulted in 75% decrease in tumor number and 88% decrease in tumor volume compared to control TSC mice. This is first evidence that drug combinations are effective in reducing size and number of kidney tumors without any toxic effect on kidney. These data will provide evidence for initiating a new clinical trial for treatment of TSC patients.

## INTRODUCTION

Tuberous Sclerosis Complex (TSC) affects around 25,000 to 50,000 individuals in the United States and about 1 to 2 million individuals worldwide, with an estimated prevalence of one in 6,000 newborns. TSC is characterized by the development of benign and/or malignant tumors in several organs including renal angiomyolipomas, facial angiofibroma, lymphangiomyomatosis, cardiac rhabdomyomas, retinal astrocytic, renal cell carcinoma, and brain subependymal giant cell astrocytomas [1–4]. Multicentric angiomyolipomas (AMLs) are much more common in patients with tuberous sclerosis than renal cell carcinoma, but may nonetheless have similar underlying genetic basis at early steps in their genesis and/or progression, specifically in the setting of tuberin deficiency [2, 3]. Renal AMLs are associated with TSC tend to be larger, bilateral, multifocal and present at a younger age compared with sporadic forms. The metastatic spread of AML cells from kidney to the lung manifests tumor growth causing a cystic destruction of the lung and lung collapse [5–7].

Loss or inactivation of *TSC1/2* genes in TSC patients results in persistent activation of Akt and mTOR (major protein kinases involved in several types of tumors), and hyperactivation of the transcription factors Hypoxia-Inducible Factors (HIF-1α and -2α) [8, 9]. Hyperactivation of HIF-1/2α in turn is positively associated with the upregulation of Vascular Endothelial Growth Factor (VEGF), a key factor in tumorigenesis and metastasis [10, 11]. Increased expression of VEGF is also associated with malignant progression and a poor treatment outcome [12]. These findings suggest that suppressing the HIF-mediated, hypoxia-induced VEGF gene pathway may be an important therapeutic strategy for the treatment of tumorigenesis in TSC. The relative contribution of HIF-1α to VEGF regulation in TSC has not yet been fully explored. The mTOR inhibitor rapamycin is also being studied as a cancer drug, both pre-clinically and clinically, but its efficacy is reported to vary with different cancer types [13–15]. On the other hand, AMP Kinase is the primary energy sensor in cells and activates tumor suppressor genes *TSC* to block HIF activity. The pharmacological activator of AMPK, 5-aminoimidazole-4-carboxamide (AICA)-riboside, or AICAR, inhibits the growth and survival of glioblastoma cells and is currently being tested as a cancer treatment [16]. Recent published data from our laboratory show that significant inhibition of mTOR by rapamycin and activation of AMPK by AICAR in several kidney tumor cells isolated from *TSC2^+/−^* mouse model [17]. We propose novel drug combinations to target the HIF/VEGF pathways to reduce tumor progression and metastasis in patients with TSC.

There are no current clinical studies using rapamycin+AICAR combination for the treatment of patients with TSC. Since rapamycin and AICAR have already been approved, and each is used separately in clinical studies (see ClinicalTrial.gov in Reference section), we propose a novel combination of rapamycin+AICAR for treatment TSC patients. Our data showed that no synergistic toxic effect of drug combinations in normal renal cells while drug combinations has stronger effect than each drug alone on inhibiting the proliferation and increased apoptosis in AML cells isolated from TSC patients and in TSC2^+/−^ and TSC2^−/−^ cells isolated from kidney of TSC2^+/−^ mice. Data from our study will provide important base-line data for clinical trials in TSC patients with kidney tumor.

## RESULTS

### Drug combinations has strong effect to induce cell apoptosis in AML cells

**Figure 1:**
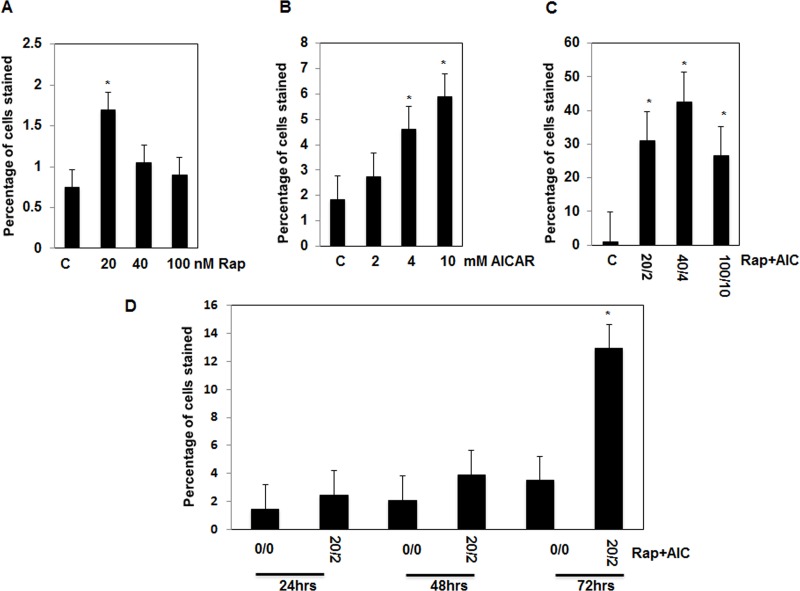

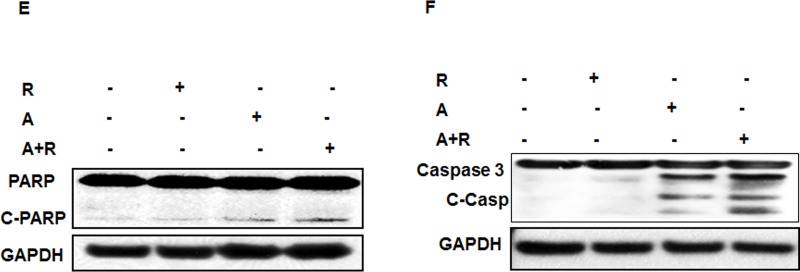
Significant increase in number apoptotic cells is dependent on drug concentration and duration of exposure in AML cells Serial concentrations of **A.,** rapamycin (0-100nM), **B.** AICAR (0-10mM) **C.,** drug combinations (0/0-2/20,4/40 and 10/100 mM/nM) in cells treated for 72hrs show that increase in number of apoptotic cells is dose-dependent using annexin V-FITC conjugated to PI by flow cytometry. In addition, treatment of the cells with drug combinations for 24, 48, and 72hrs **D.,** show that increase in number of apoptotic cells is time dependent. Apoptotic data was confirmed in cells by measuring apoptotic protein expression. Lysates from cells treated with rapmycin (20 nM), AICAR (2 mM), rapamycin+AICAR (20 nM/2 mM) for 72hrs were subjected to Western blot analysis to measure PARP and caspase 3 cleavages. **E.** & **F.** Significant increase was detected in cleavage of PARP at 85 KDa and caspase 3 at 22, 17 and 11KDa in AML cells treated with drug combinations compared to cells treated with each drug alone for 72hrs. GAPDH was used as a loading control. Data represent means±SE (*n* = 4). Significant difference from control tissues is indicated by * *P* < 0.01.

To test the effective dose of each drug or the synergistic effect of drug combinations on cell apoptosis, cells treated with serial concentrations of AICAR (0-10mM) or rapamycin (0-100nM) or combination of both drugs (2/20, 4/40, 10/100, mM/nM) for 72 hrs. AML cells treated with rapamycin or AICAR show increase in number of apoptotic cells, which is dose dependent with maximum of 3-fold with AICAR (10mM) and 2 fold with rapamycin (20nM) compared to non-treated cells measured by annexin V assay (Figure 1A & 1B). On the other hand, the most effective low dose of combined drugs (2/20, mM/nM) showed 10-fold increase in number of apoptotic cells compared to non-treated cells (Figure 1C). In addition, cells were treated with drug combinations (2 mM/20 nM, AICAR/Rapa) for different time points (24, 48 and 72 hrs) show that increase in cell apoptosis is associated with increase exposure time of the cells to drugs (Figure 1D). Furthermore, we confirmed the increase in apoptosis proteins in cells treated with each drug and drug combinations by measuring cleavage of PARP at 85 kDa and Caspase 3 at 22, 17, 11 kDa products (Figure 1E & 1F), confirming that the combination of drugs has strongest effect on increasing the apoptosis cascade pathway compared to cells treated with each drug alone.

### Drug combinations significantly decreased cell proliferation in AML cells

**Figure 2:**
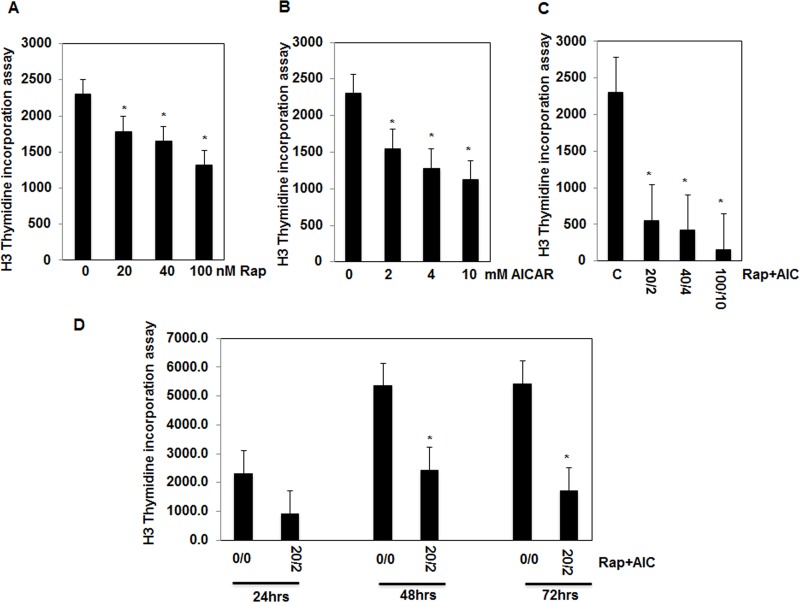

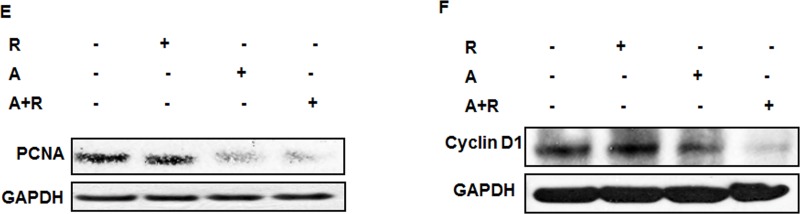
Decrease cell proliferation in response to drug combinations is dependent on the drug concentration and duration of exposure in AML cells Serial concentrations of **A.,** rapamycin (0-100nM), **B.,** AICAR (0-10mM) **C.,** drug combinations (0/0-2/20,4/40 and 10/100 mM/nM) in cells treated for 72hrs showed that decrease in cell proliferation is dose-dependent using ^3^H-thymidine incorporation assay. In addition, treatment of the cells with drug combinations for 24, 48, and 72hrs **D.,** showed that significant decrease in cell proliferation is time dependent. Cell proliferation data was confirmed by measuring proliferative protein expression. Lysates from cells treated with rapmycin (20nM), AICAR (2mM), rapamycin+AICAR (20nM/2mM) for 72hrs were subjected to Western blot analysis to measure PCNA and cyclin D1. **E.** & **F.** Significant decrease in expression of PCNA and cyclin D1 was detected in cells treated with single drug while abolishment of expression of both proteins detected in cells treated with drug combinations providing evidence of the synergistic effect of drug combinations on reducing cell proliferation. GAPDH was used as a loading control. Data represent means±SE (*n* = 4). Significant difference from control tissues is indicated by * *P* < 0.01.

Since cell proliferation is necessary for tumor progression, we tested the effect of each drug or drug combinations on cell proliferation. AML cells were treated with serial concentrations of AICAR (0-10mM) or rapamycin (0-100nM) or combination of both drugs (2/20, 4/40, 10/100, mM/nM) for 72 hrs. The [^3^H]-Thymidine incorporation assay performed in all treated cells showed that the decrease in cell proliferation is dose dependent for each drug alone. Cells treated with rapamycin or AICAR show a dose dependent decrease in number of proliferative cells, with maximum of 1-fold decrease at highest dose of each drug compared to non-treated cells (Figure 2A & 2B). On the other hand, the decrease in the cell proliferation show strong dose dependent effect from 2/20, 4/40 and 10/100 mM/nM AICAR/rapamycin, (8-9 fold decrease), with the most effective lowest dose of combined drugs of 2mM/20nM compared to non-treated cells (Figure 2C). In addition, cells were treated with drug combinations (2mM/20nM, AICAR/Rapa) for different time points (24, 48 and 72 hrs) show that decrease in cell proliferation is associated with increase in the exposure time of the cells to drugs (Figure 2D). Furthermore, we confirmed the decrease in cell proliferative proteins in both cells treated with each drug and most effectively in cells treated with drug combinations measured by protein expression of PCNA and cyclin D1 (Figure 2E & 2F). Significant decrease in both proliferative proteins expression was observed in cells treated with drug combinations compared to each drug alone (Figure 2E & 2F). These data suggest that combined drug has strong synergistic effect compared to each drug alone to decrease cell proliferation and slow the tumor progression.

### Drug combinations is more effective to block survival kinase (Akt) and to abolish HIF expression in AML cells

**Figure 3:**
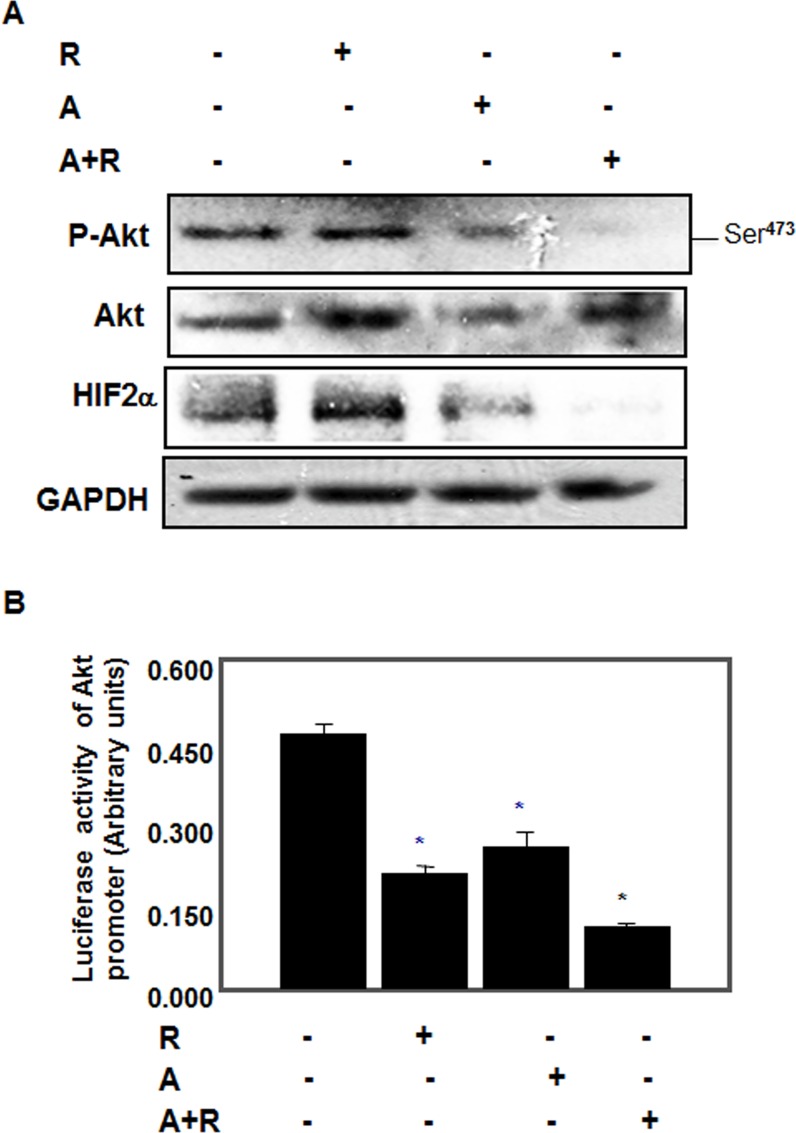

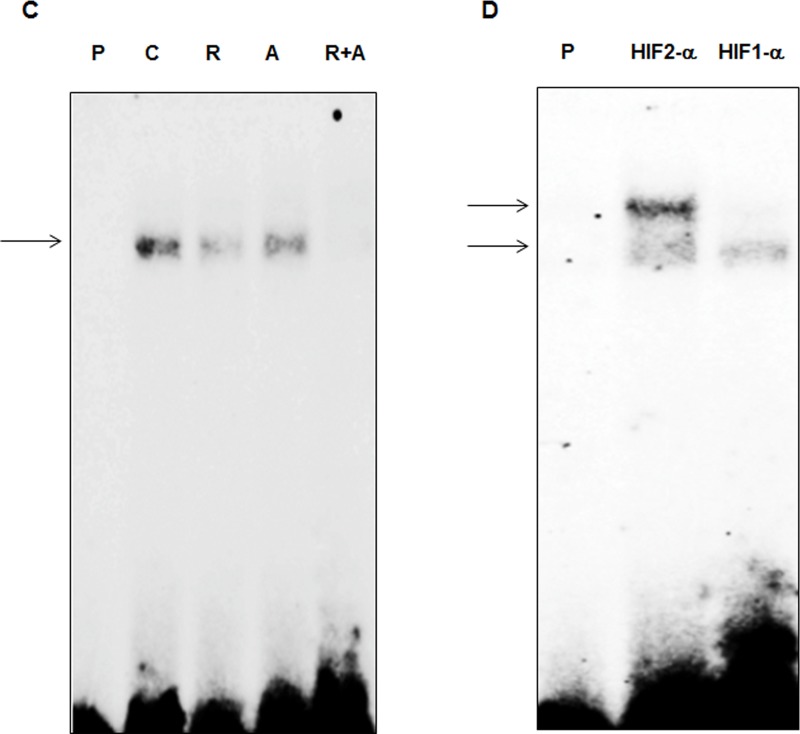
Drug combinations abolished Akt survival kinase and blocked binding of HIF-2α to Akt promoter in AML cells Cells were treated with rapamycin (20 nM) or AICAR (2 mM) or drug combinations (20 nM rap/2 mM AICAR) for 72 hrs. Cell lysates were subjected to Western blot analysis to measure p-Akt, HIF-2α expression. **A.** Significant decrease in p-Akt, HIF-2α expression in cells treated with single drug while synergistic effect of drug combinations showed complete abolishment in HIF-2α as well as in phosphorylation of Akt at Ser^473^ expression compared to cells treated with single drug and control cells. GADPH was used as a loading control. Further evidence of the effect of drug combinations on HIF-2α function was measured by luciferase assay. **B.** The synergistic effect of drug combinations showed more than 70% decrease in the promoter activity of HIF-2α compared to control cells using the Luciferase Reporter Assay System normalized by Renilla activity and measured by a luminometer. Data represent means±SE (*n* = 4). Significant difference from control cells is indicated by * *P* < 0.01. A combination of drugs blocks binding of HIF2α to the Akt promoter element. **C.** EMSA analysis of DNA probe corresponding to the putative HIF-2α binding site in the Akt promoter was performed. Labeled probes were incubated with nuclear extracts isolated from AML cells treated with rapamycin, AICAR or rapamycin+AICAR showed significant decrease in binding of HIF-2α into the Akt promoter. **D.** Specificity of binding of HIF-α to Akt promoter was confirmed by adding HIF1α or HIF-2α antibody to the reaction mixture. Data confirmed that HIF-2α not HIF-1α as a part of DNA-protein complex. These data provide new evidence that HIF2α is a major transcription factor that regulates cell survival kinase Akt.

Hyperactivation of Akt and mTOR, the major protein kinases were detected in several types of tumors. In addition, overexpression of the transcription factors Hypoxia-Inducible Factors HIF-1α and -2α was linked to tumor progression of kidney tumorigenesis. Next, we tested the effect of each drug or drug combinations on phosphorylation of Akt, and protein expression of HIF-2α in renal AML cells. Cells were treated with rapamycin (20 nM) or AICAR (2 mM) or drug combinations (20 nM rap/2 mM AICAR) for 72 hrs. Cells treated with single drug show significant decrease in p-Akt) compared to control (Figure 3A). On the other hand, combination of drugs nearly abolished phosphorylation of Akt at Ser^473^ (Figure 3A) and abolished HIF-2α protein expression (Figure 3A) indicating that the combination of the drug has more effect than each drug alone in blocking activation of Akt/HIF-2α pathway.

### Drug combinations is more effective on decreasing Akt promoter activity in AML cells

Akt promoter reporter plasmid was transfected and *Renilla* reporter plasmid (pRL-null) as transfection control into AML cells as described in M&M section. Cells were treated with rapamycin (20 nM) or AICAR (2 mM) or drug combinations (20 nM rap/2 mM AICAR) for 72 hrs. Cells were harvested for firefly and Renilla luciferase assay using Dual-Luciferase Reporter assay kit. Data in Figure 3B show that each drug treatment has significant effect on decrease luciferase activity of Akt promoter activity compared to control cells. On the other hand, treatment of the cells with drug combinations showed close to 70% decrease in the Akt promoter activity compared to control cells suggesting that the synergistic effect of the combined drugs is sufficient to block the promoter activity of the major transcription factor that involve in kidney cancer. These data suggest that drug combinations have a strong synergistic effect to block activation of Akt survival kinase as well as accumulation of HIF-2α through activation of AMPK and inhibition of mTORC1.

### Drug combinations block binding HIF2α to Akt promoter in AML cells

To provide another evidence of the effect of combined drugs on HIF-2α function, we examined the effect of each drug and drug combinations on binding of HIF-2α to the putative binding sequence in the Akt promoter using nuclear extracts from AML cells. Nuclear extracts from control or cells treated with 2mM AICAR or 20 nM rapa or 2-mM/20nM AICAR/rapa were used to perform electrophoretic mobility shift assay (EMSA). Cells treated with single drug show no significant changes compared to control cells while cells treated with both drugs almost abolished the binding of the HIF-2α to the putative *Akt1* promoter (Figure 3C). The specificity of HIF-2α as a part of DNA-protein complex was tested by pre-incubating the nuclear proteins with HIF1α or HIF-2α antibody. Data in Figure 3D show that the DNA/protein complex shifted when only HIF-2α antibody was added in the reaction suggesting that HIF2α is major transcription factor that binds to Akt promoter to activate cell survival. Taken together, these data provide new evidence that HIF2α is a major transcription factor that regulates cell survival kinase Akt.

### Drug combinations significantly decreased cell migration and cell invasion of AML cells

**Figure 4:**
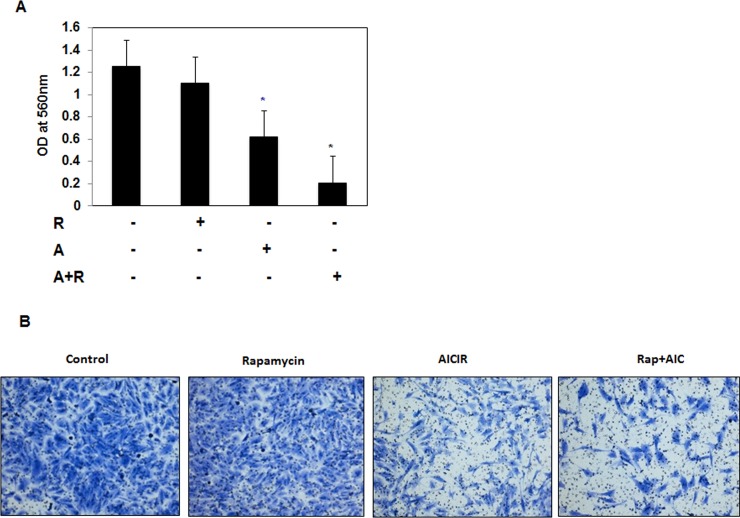

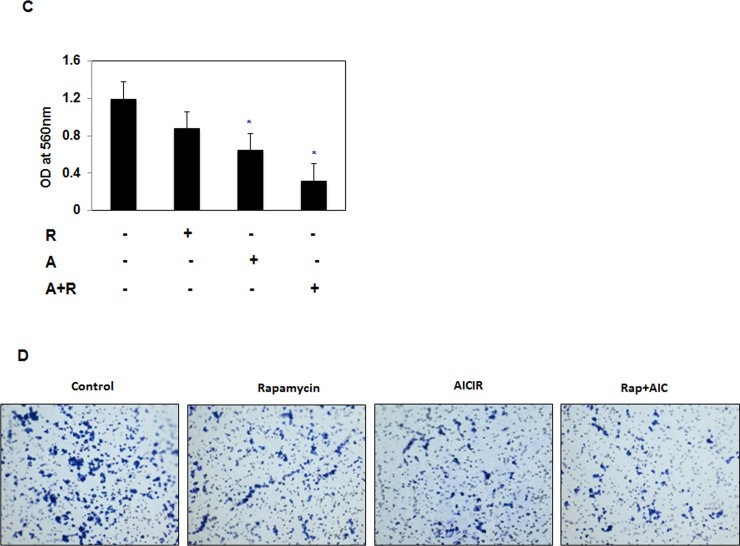
Drug combinations significantly decreased cell migration and cell invasion of AML cells AML cells treated with rapamycin (20nM) or AICAR (2mM) or drug combinations (20nM rap/2mM AICAR) for 72 hrs were added into the upper well chamber of 96-wells plate. Data in **A.** & **B.** show that cells treated with drug combinations has significant low number of migrated cells and **C.** & **D.** invaded cells compared to cells treated with single drug or control cells. The total number of migrated and invaded cells was counted using counting software and the images of migrated or invaded cells were taken using Nikon light inverted microscope. Significant difference from control cells is indicated by * *P* < 0.01.

It is known AML cells have migration and invasion character to promote metastasis. Next, we tested the effect of each drug or drug combinations on cell invasion and cell migration. AML cells treated with rapamycin (20nM) or AICAR (2mM) or drug combinations (20nM rap/2mM AICAR) for 72 hrs showed different patterns of cell migration and invasion. Data in Figure 4A & 4B show that cells treated with drug combinations has significant low number of migrated cells compared to cells treated with single drug or control cells. We tested whether each drug alone or drug combinations will have more effect on cell invasion. Data in Figure 4C & 4D show that cells treated with drug combinations has significant low number of invaded cells compared to cells treated with single drug or control cells. These data suggest that the synergistic effect of the drug combinations on preventing the cells migration and invasion will have important role in preventing tumor metastasis.

### Drug combinations significantly increased cell apoptosis in fresh primary TSC2^+/−^ and TSC^−/−^ cells isolated from kidney of TSC2^+/−^ mice

**Figure 5:**
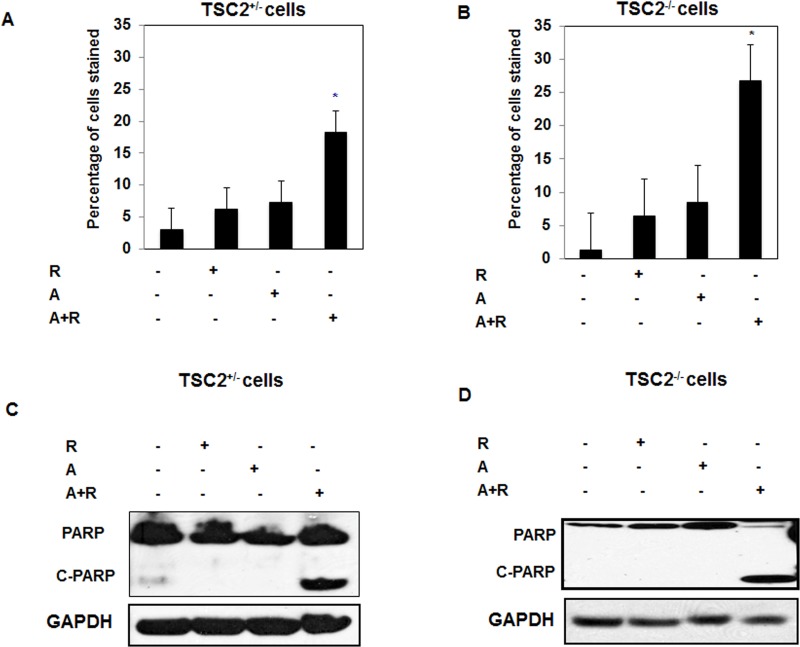
Synergistic effect of drug combinations significantly increased cell apoptosis, decreased cell proliferation, blocked Akt and decreased HIF-2α expression in primary TSC2^+/−^ and TSC^−/−^ cells **A.** & **B.** Single drug treatment (Rap, 20nM), AICAR (20mM) and drug combinations (2/20 mM/nM) show significant increased in number of apoptotic cells in cells treated with drug combinations using annexin V-FITC conjugated to PI by flow cytometry in both primary TSC2^+/−^ and TSC^−/−^ cells. **C.** & **D.** Lysates from cells treated with rapmycin (20nM), AICAR (2mM), AICAR+rapamycin (2mM/20nM) for 72hrs showed significant increase in cleavage of PARP at 85 KDa in TSC2^+/−^ and TSC^−/−^ cells treated with drug combinations compared to cells treated with each drug alone. GAPDH was used as a loading control. Data represent means±SE (*n* = 4). Significant difference from control cells is indicated by * *P* < 0.01.

We tested the effect of each drug and drug combinations in TSC cells isolated from kidney of TSC mice to provide *in vitro* evidence before starting *in vivo* experiment using TSC mice. Fresh primary cells isolated from normal (TSC2^+/−^) and kidney tumor (TSC2^−/−^) tissues of mice were used to confirm the effect of treatment with drug combinations versus single drug alone. Data in Figure 5A & 5B show 6-fold and 14 fold increase in number of apoptotic cells in TSC2^+/−^ and TSC2^−/−^ respectively after treatment with combined drugs compared to non-treated cells. In addition, cleavage of apoptotic protein, PARP at 85 kDa was significantly increased in treated cells with drug combinations compared to cells treated with each drug alone for 72hrs (Figure 5C & 5D). These data suggest that drug combinations are more effective than single drug in increasing number of apoptotic cells.

### Drug combinations significantly decreased cell proliferation in fresh primary TSC2^+/−^ and TSC^−/−^ cells isolated from kidney of TSC2^+/−^ mice

**Figure 6:**
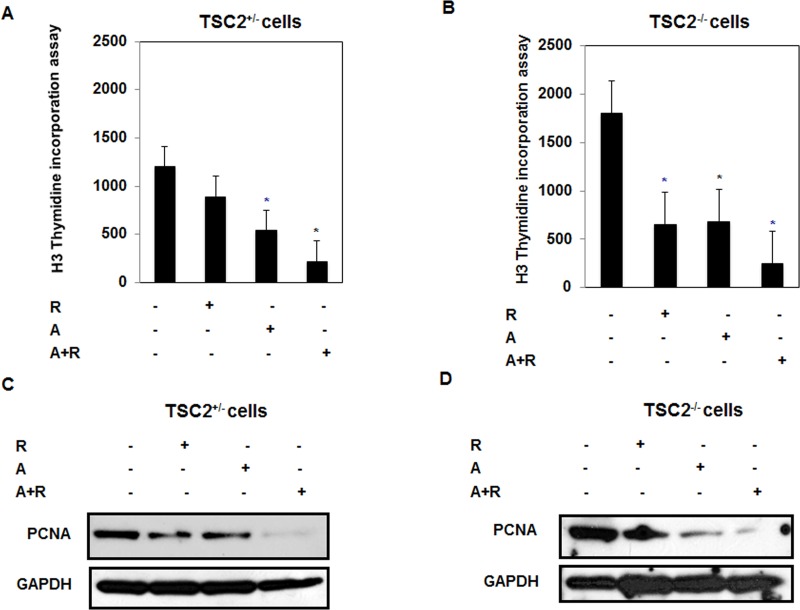
Decrease in cell proliferation by drug combinations is dependent on the drug concentration and duration of exposure in primary TSC2^+/−^ and TSC^−/−^ cells **A.** & **B.** Single drug treatment (Rap, 20nM), AICAR (20mM) and drug combinations (2/20 mM/nM) showed significant decrease in cell proliferation using ^3^H-thymidine incorporation assay in both primary TSC2^+/−^ and TSC^−/−^ cells. Cell proliferation data was confirmed by measuring proliferative protein expression. Lysates from cells treated with rapmycin (20nM), AICAR (2mM), rapamycin+AICAR (20 nM/2 mM) for 72hrs were subjected to Western blot analysis to measure PCNA and cyclin D1. **C.** & **D.** Significant decrease in expression of PCNA was detected in cells treated with single drug while abolishment of expression of both proteins detected in cells treated with drug combinations providing evidence of the synergistic effect of drug combinations in reducing cell proliferation. GAPDH was used as a loading control. Data represent means±SE (*n* = 4). Significant difference from control cells is indicated by *P< 0.01.

The synergistic effect of drug combinations and each drug treatment on reducing cell proliferation was tested in fresh primary cells of TSC2^+/−^ and TSC^−/−^ mice. Data in Figure 6A & 6B show that drug combinations treatment resulted in 5 fold and 6 fold decrease in cell proliferation in TSC2^+/−^ and TSC^−/−^ respectively compared to non-treated cells. In addition, abolishment of expression of PCNA in TSC2^+/−^ cells treated with drug combinations compared to non-treated cells or single drug treatment (Figure 6C). While significant decrease in PCNA was observed in TSC2^−/−^ cells treated with drug combinations (Figure 6D). These data suggest that drug combinations are more effective on slowing tumor progression by decreasing cell proliferation and increasing cell apoptosis.

### Synergistic effect of drug combinations abolished Akt phosphorylation and HIF2α expression in fresh primary TSC2^+/−^ and TSC^−/−^ cells isolated from kidney of TSC2^+/−^ mice

**Figure 7:**
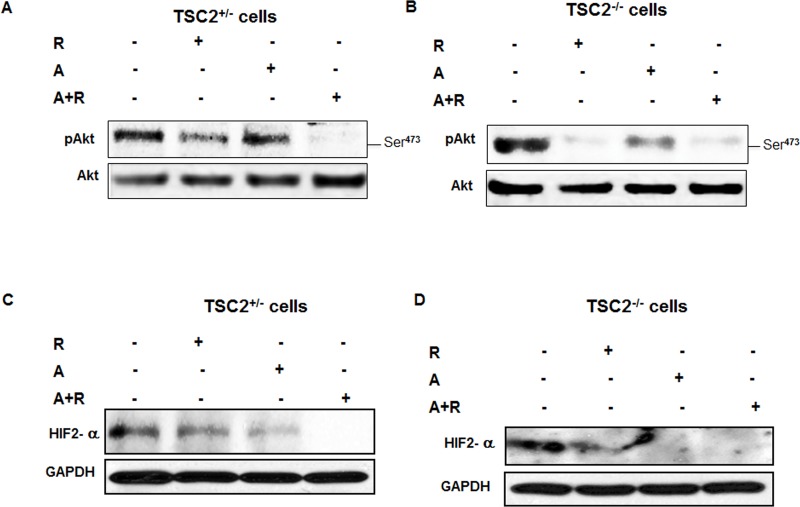

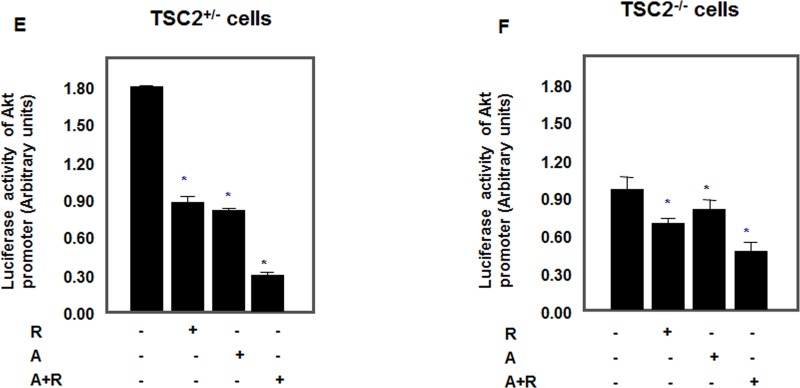
Abolished p-Akt and HIF-2α expression in cells treated with drug combinations **A.**-**D.** TSC2^+/−^ and TSC^−/−^ cell extracts of treated cells with single drug or drug combinations were subjected to Western blot analysis. Abolished phosphorylation of Akt at Ser^473^ and HIF-2α protein expression in TSC2^+/−^ cells and significant decrease in p-Akt and HIF-2α expression in TSC^−/−^ cells compared to non-treated cells. GADPH was used as a loading control. **E.** & **F.** The synergistic effect of drug combinations showed more than 84% and 50% decrease in the promoter activity of HIF-2α in TSC2^+/−^ and TSC^−/−^ cells compared to control cells. The Luciferase Reporter Assay was normalized by Renilla activity and measured by a luminometer. Data represent means±SE (*n* = 4). Significant difference from control cells is indicated by * *P* < 0.01.

Abolishment of Akt phosphorylation at Ser4^73^ expression was detected in TSC2^+/−^ and TSC^−/−^ cells treated with drug combinations compared to cells treated with single drug and control cells (Figure 7A-7D). In addition, rapamycin and AICAR significantly decreased phosphorylation of Akt and HIF2α expression compared to non-treated TSC2^+/−^ and TSC^−/−^ cells (Figure 7A-7D). Moreover, the effect of each drug or drug combinations on Akt promoter activity was tested in TSC2^+/−^ and TSC^−/−^ primary cells. Data in Figure 7E & 7F show significant decrease in Akt promoter activity measured by luciferase compared to non-treated cells, while sharp decrease in Akt promoter activity was observed in cells treated with drug combinations in TSC2^+/−^ and TSC^−/−^ cells (Figure 7E & 7F). These data provide a new evidence of synergistic effect of drug combinations on blocking AKT/HIF-2 pathway to decrease tumor progression. These data also provide strong evidence that drug combinations will be more effective than each drug alone to treat TSC2^+/−^ mice.

### No toxicity in kidney of TSC2^+/−^ mice treated with single drug or drug combinations

**Figure 8:**
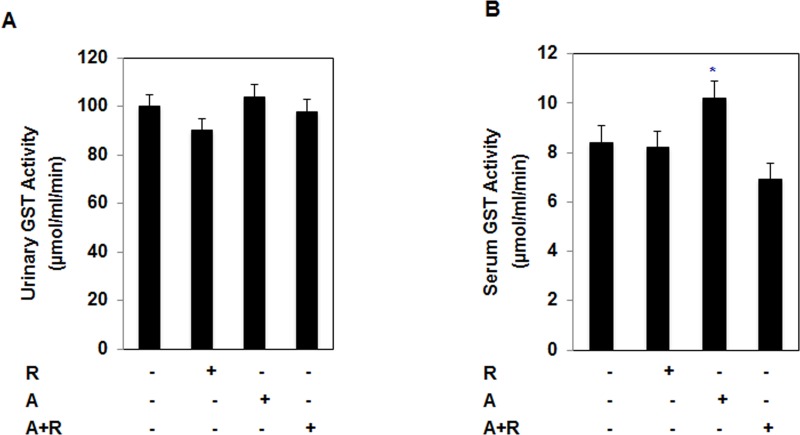
Drugs alone and in combination show no nephrotxoicity in treated mice Renal injury was determined by measuring the **A.,** urinary and **B.,** serum excretion of GST in mice following treatment with rapamycin or AICAR or combination of rap + AIC for 4 weeks. AICAR treatment showed slight increase in GST activity in serum but not major changes between 4 groups of mice indicating no toxicity of treatment with single drug or drug combinations. Values represent the mean ± SE (*n* = 6) and GST activity represent as μmol/ml/min. Significant difference from control mice is indicated by * *P* < 0.01.

To test the side effect of drug toxicity on animals during the injection, urinary and serum excretion of glutathione-*S*-transferase (GST) as an indicator of the loss of cell membrane integrity animal was determined in all 4 experimental groups. Urine was collected a day before sacrificing the mice and serum collected during sacrificing the mice. Data in Figure 8A & 8B show that no significant changes in the levels of urine and serum of GST activity between mice treated with single drug or drug combinations compared to control group of mice. Only AICAR treatment shows increase in serum GST compared to control and other groups. These data provide new evidence of the safety of drug combinations as a novel direction for future treatment of kidney cancer.

### Drug combinations are more effective on reducing tumor size and number in TSC^+/−^ mice

**Figure 9:**
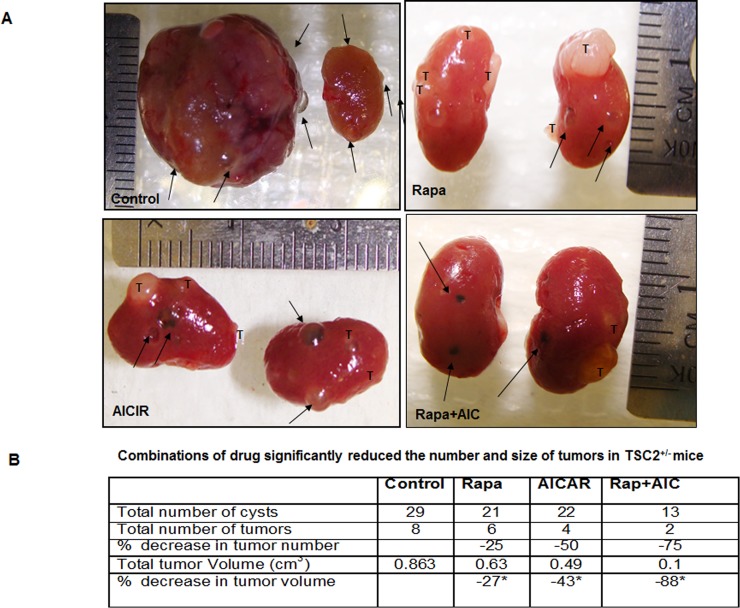
Synergistic effect of drug combinations resulted in significant decrease in kidney tumor size and number in TSC2^+/−^ mice **A.** Macroscopic view of all mice kidneys was examined for cysts, number and size of tumors. Tumor size was measured in three axes as cm^3^ (length, width, and height). **B.** Treatment with rapamycin resulted in significant decrease in cysts number, 25% decrease in tumor number and 27% decrease in tumor volume compared to control mice. AICAR treatment showed 50% decrease in tumor number and 43% in tumor volume compared to non-treated group. Major decrease in tumor size and number was noticed in mice treated with drug combinations and resulted in 75% decrease in tumor number and 88% decrease in tumor volume compared to non-treated mice. Cysts marked with arrow and tumor marked with T. Significant difference from control mice is indicated by * *P* < 0.01.

We further investigated the synergistic effect of drug combinations on reducing kidney tumor size *in vivo*. TSC2^+/−^ mice at age of 12 months were divided to 4 groups, control (mice were injected with an equal amount of DMSO). Group 2, (mice injected with 2 mg/kg body weight rapamycin), group 3 (mice injected with 250mg/kg body weight AICAR) and group 4 (mice injected with same doses of rapamycin (2mg/kg) and AICAR (250mg/kg). Animals were euthanized after treatment and kidneys were removed rapidly to measure tumor size by two independent observers blinded to the experimental conditions. Image of kidney from all 4 groups of mice in Figure 9A show the differences in tumor sizes within 4 groups of mice. Data in Figure 9B show that treatment with rapamycin resulted in significant decrease in cysts number, 25% decrease in tumor number and 27% decrease in tumor volume compared to control mice. AICAR treatment showed 50% decrease in tumor number and 43% in tumor volume compared to non-treated group. Major finding from our study that treatment with drug combinations resulted in 75% decrease in tumor number and 88% decrease in tumor volume compared to non-treated mice. These data provide a novel role of drug combinations on decreasing tumor size and number and will be helpful to develop a clinical trail study in TSC patients.

### Drug combinations are more effective to block cell proliferation and increase cell apoptosis in kidney tumor

**Figure 10:**
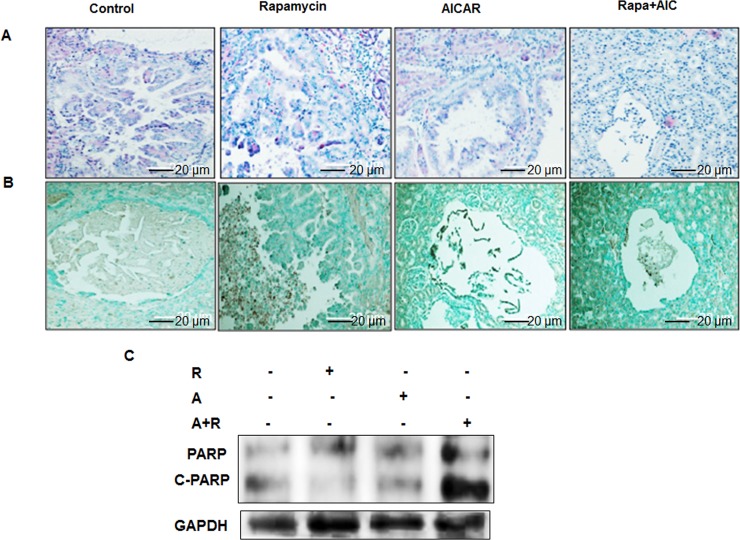

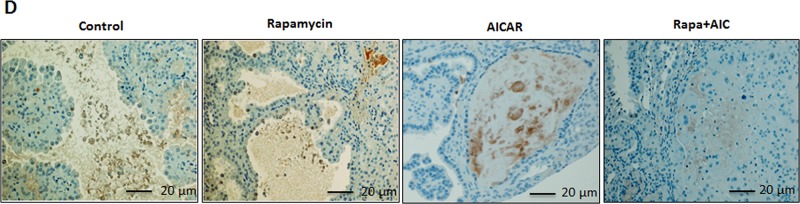

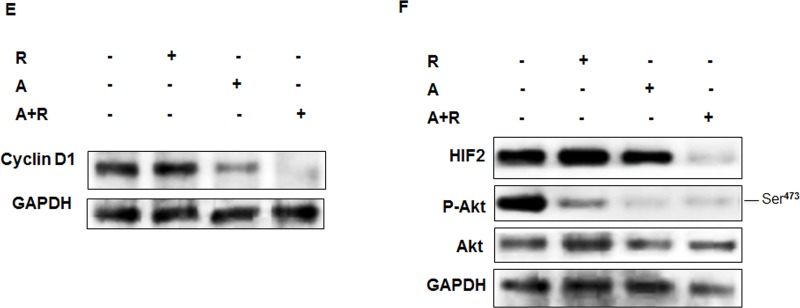
Synergistic effect of drug combinations strongly increased cell apoptosis, decreased cell proliferation and decreased p-Akt/HIF-2α expression TUNEL assay was performed in kidney sections of 4 groups of mice and pictures taken by light microscope. **A.** H&E staining of kidney from all 4 groups of mice show the differences in tumor sizes within 4 groups of mice. **B.** Stained nucleus for TUNEL show significant increase in number positive cells in mice treated with drug combinations and less in mice treated with single drug compared to control mice group. In addition, tumor kidney homogenates from all 4 mice groups was subjected to Western blot analysis. **C.** Apoptosis proteins including cleavage of PARP was measured in kidney sections of mice treated with drug combinations and mice treated with single drug and control mice group. **D.** Proliferative proteins expression of Ki67 showed that drug combinations significantly decrease Ki67 staining in nucleus in kidney tumor compared to tumor from mice treated with single drug and control mice group. **E.** Western blot analysis was performed in tumor homogenates from 4 groups of mice show significant decrease in cyclin D1 in mice treated with drug combinations compared to mice treated with single drug and control mice group. **F.** Data of Western blot showed that drug combinations nearly abolished p-Akt and significantly decreased expression of HIF-2 protein expression in tumor tissues compared to tumor from mice tested with single drug and control mice group.

H&E staining showed the size and histology of tumor in kidney sections from 4 groups of mice (Figure 10A). TUNEL assay was performed in kidney sections from each mouse to test the effect of treatment with single drug versus drug combinations of cell apoptosis. Kidney sections were examined using light microscopy and number of apoptotic cells in mice treated with drug combinations and less in group treated with single drug compared to control mice group (Figure 10B). These data was confirmed in tumor kidney homogenates from all 4 mice groups. Strong cleavage in PARP at 85 kDa in mice treated with drug combinations compared to mice treated with single drug and control mice group (Figure 10C). On the other hand, cell proliferation staining using Ki67 proliferative marker was performed in kidney sections from each group of mice. Data in Figure 10D show significant decrease in number of stained nucleus with Ki67 in kidney tumor mice treated with drug combinations and less in mice treated with single drug compared to control mice group. These data was also confirmed in tumor kidney homogenates from all 4 groups using proliferative protein markers (cyclin D1) by Western blot analysis. Data in Figure 10E show that drug combinations have strong effect in decreasing cyclin D1 expression compared to mice treated with single drug and control mice group. These data suggest that the synergistic effect of drug combinations have strong impact to slow the progression of tumor by decreasing cell proliferation and increasing cell apoptosis.

### Drug combinations has strong effect on inhibiting cell survival kinase and decreasing HIF-2α expression

Akt is the major cell survival kinase therefore identifying a drug(s) can slow cancer cell survival will be important to control tumor progression. In addition, hyperactivation of HIF-1/2α is a key factor in tumorigenesis. Tumor tissue homogenates from all 4 groups of mice were subjected to Western blot analysis. Data in Figure 10F show that P-Akt at Ser^473^ and HIF-2α expression is significantly decreased in mice treated with drug combinations compared to mice treated with single drug and control mice group. Taken together these data provided a strong evidence of the synergistic effect of drug combinations on reducing tumor progression in TSC mice through regulation cell apoptosis and cell proliferation pathways.

## DISCUSSION

Rapmaycin has been used for treatment several types of cancer and AICAR have been approved in clinical studies but no clinical trial registered for using combination of both drugs for treatment of TSC patients. Our data tested the synergistic effect of the combination of rapamycin+AICAR for treatment of kidney tumorigenesis in AML human and in TSC2^+/−^ and TSC^−/−^ fresh tubular cells isolated from normal and tumor kidney of TSC2^+/−^ mouse and in TSC2^+/−^ mouse model. Drug combinations have strong synergistic effect to decrease cell proliferation, increase cell apoptosis and abolish Akt/HIF2α expression in AML, TSC2^+/−^ and TSC^−/−^ cells. We have identified a putative HIF-1/2α binding site in the *Akt1* promoter. In addition, drug combinations effectively abolished the binding of HIF-2α to the putative *Akt1* promoter providing new evidence that HIF-2α is a major transcription factor involved in activation cell survival kinase (Akt). Importantly, the synergistic effect of the drug combinations shows important influence on decreasing cells migration and invasion compared to single drug treatment suggesting that combined drugs has a major effect in preventing tumor progression and metastasis. Our major *in vivo* data finding that treatment of TSC mice with drug combinations resulted in 75% decrease in tumor number and 88% decrease in tumor volume compared to control TSC mice.

One of the major anticancer drugs side effects is drug toxicity to kidney. Data from injection of single drug or drug combinations into mice for 4 weeks did not show any significant changes in the levels of GST activity as an indicator of early marker for renal dysfunction [25] in mice treated with single drug or drug combinations compared to control mice group suggesting that drug combinations may be useful for future treatment of TSC patients. Drug combinations shows significant increase in cell apoptosis, decrease in cell proliferation, sharp decrease in survival kinase (Akt) and decrease in major transcription factor HIF-2α in tumor tissues compare to single drug and control mice group.

The mammalian target of rapamycin (mTOR) plays a central role in regulating cell growth, proliferation and survival, in part by the regulation of translation initiation [28–30]. In addition, mTOR signaling pathways are constitutively activated in many types of human cancer [30]. It is known that mTORC2 promotes activation of the cell survival protein by phosphorylation of Akt at residues Ser^473^ and other growth factors [9]. Targeting mTOR is emerging as an important approach in cancer therapeutics. Early clinical trials show that kidney tumours regress in response to treatment with rapamycin and other mTOR inhibitor including everolimus, a direct-target of inhibition of mTORC1 [31, 32]. In addition, activation AMPK by AICAR show significant increase in cell apoptosis and decrease in cell proliferation by targeting Akt/HIF2-α in AML, TSC2^+/−^ and TSC^−/−^ cells.

AMPK has been recognized as an important upstream signaling intermediate intimately involved in the regulation of the mTOR pathway. Our data show that AICAR has significant effect in reducing HIF-2α and cell invasion of AML cells compared to control cells. Our previous data show that activation of AMPK by AICAR results in decreased p70S6K phosphorylation and increased DNA repair expression protein OGG1 expression and rapamycin activates AMPK leading to increased OGG1 protein expression in renal cancer cells [17]. *in vivo* and *in vitro* observation showed that blocking mTOR1 and mTORC2 activation results in prevention of tumor progression in TSC^+/−^ mice. In accordance with our data, similar recent findings show that new mTOR kinase inhibitor (WYE-687) increased apoptosis and blocked activation of both mTORC1 and mTORC2 through the feedback activation of p-Akt in renal cancer cells as well as the oral administration of WYE-687 potently suppressed tumor growth in nude mice injected with renal cancer cells [33]. These findings suggest that suppressing the HIF-2 may be an important therapeutic strategy for the treatment of tumorigenesis in TSC patients. Our data show that blocking mTORC1 by rapamycin increases cell apoptosis and significantly decreases cell proliferation targeting Akt/HIF2-α pathway to influence number of migrated and invaded AML cells. Major finding of our work show that treatment of the mice with drug combinations resulted in 75% decrease in tumor number and 88% decrease in tumor size compared rapamycin (25% in tumor number and 27% in tumor size) and AICAR (50% decrease in tumor number and 43% decrease in tumor size) suggesting that the drug combinations are more effective in reducing tumor size compared to each drug alone. For our knowledge, there is none clinical studies of AMPK activator (AICAR or metformin) that used for TSC-associated AML but AICAR or metformin have been used in clinical trial for kidney cancer. Data from our study provided the first evidence of novel approach of the effective of drug combination of AICAR+Rapamycin on blocking tumorigenesis in TSC-animal model and also the safety of using drug combinations without any toxicity in kidney.

In summary, we showed that single and combined drugs have no significant effect on the body weight and no toxicity to the kidney of TSC2 mice. Treatment with drug combinations has strong effect in inhibiting cell proliferation and increasing cell apoptosis to block cell survival kinase (Akt) and abolish HIF-2 expression in AML, TSC2^+/−^ and TSC^−/−^ cells and in TSC2^+/−^ mouse model. Major finding of our work is that low dose of drug combinations reduced 88% of kidney tumor size compared to control mice and these finding provide a new evidence of the strong effect of drug combinations in reducing kidney tuomorigenesis. Taken together *in vitro* and *in vivo* data show new finding of the role of drug combinations in reducing kidney tumor progression in TSC mouse and new evidence that drug combinations has more benefit to treat TSC patients than single drug alone. Our data provided a new mechanism of preventing kidney tumor progression without side effect of drug combinations in the treated mice. These data provide *in vivo* evidence to initiate a clinical trail for treatment of TSC patients with kidney tumors.

## MATERIALS AND METHODS

### Cell culture

Angiomyolipoma (AML) cells derived from human kidney of TSC patient were generously provided by Dr. Elizabeth Henske (Harvard Medical School, MA) [18]. Mouse tubular cells were isolated from normal (TSC2^+/−^) and tumor (TSC2^−/−^) kidney mouse and cultured as previously described [19]. AML, TSC2^−/−^ and TSC2^+/−^ cells were grown in DMEM supplemented with 10% FBS at 37°C in a humidified atmosphere of 5% CO_2_.

### Cells treatment

AML cells were treated with serial concentrations of AICAR (0, 2, 4, 10 mM) [16] or rapamycin (0, 20, 40, 100 nM) [17] or combination of AICAR and rapamycin (0/0, 2/20, 4/40, 10/100 mM/nM, AICAR/rapamycin) for 72 hrs. Cells were also treated with drug combinations (2 mM/20 nM, AICAR/Rapa) for 24, 48 and 72 hrs. AICAR was obtained from Cayman Chemical (Ann Arbor, MI) and rapamycin from Sigma-Aldrich (St. Louis, MO).

### Thymidine assay

Cell proliferation was measured using ^3^H-thymidine incorporation assay. Cells were plated into 24-well dish and treated with serial concentrations of rapamycin or AICAR or drug combinations as described above for 72 hrs. At the time of treatment, cells were pulsed with ^3^H thymidine. After the treatment period, medium was removed and the cells were fixed in 5% TCA for 10 min. Cells were then washed twice in 5% TCA for 10 min each at room temperature to remove unincorporated thymidine. Cells were lysed in 750 μl of 0.25N NaOH and 0.1% SDS. The cell lysates (500 μl) were transferred into vials containing 5 ml scintillation solution and 50 μl of 6N HCl. ^3^H-thymidne incorporated was counted in a scintillation counter.

### Annexin V assay

Apoptosis was detected using Annexin V-FITC detection kit (EMD Millipore, San Diego, CA) as previously described [20]. Briefly, cells were trypsinized, counted using hemocytometer, and 200,000 cells were taken for assay. Cells were centrifuged at 1000Xg for 5 min, washed in PBS, suspended in 400 μl of 1X binding buffer containing 1.25 μl of Annexin V-FITC. Cells were incubated for 20 min at room temperature in dark. After incubation, cells were centrifuged, suspended in 400 μl of 1X binding buffer containing 10 μl of propidium iodide and incubated for 10 min in dark. Apoptotic cells were analyzed by flow cytometry (Becton-Dickinson, Rutherford, NJ).

### Western blot analysis

Cell and tissue lysates were prepared in RIPA lysis buffer using a dounce homogenizer as previously described [21]. Protein concentration was determined using Bradford reagent [22]. An aliquot of 30 to 50 μl of lysate was electrophoresd on a SDS-polyacrylamide gel. Western blotting was carried out as previously described [21]. Phospho-Akt at Ser^473^, Akt, PCNA, cyclin D1, HIF1/2-α and Caspase 3 antibodies were purchased from Cell Signaling Technology (Danvers, MA); PARP and GADPH antibodies were obtained from Santa Cruz Biotechnology. Expression of each protein was quantified by densitometry using National Institutes of Health image 1.62 software and normalized to a loading control.

### Generation of Akt promoter-luciferase reporter plasmid

The *Akt* promoter region (−1 to −1991) containing a potential binding HIF-2α site was cloned into luciferase reporter vector (pGL3). The primers used were: Forward primer: 5′-GGTGCCCGAAGCTTCCGCGACGCT-3′, Reverse primer: 5′- GGCCACAGAGCTCCTCAGCAGTCCCAG-3′. Act promoter reporter plasmid was used to determine the transcriptional activity of the HIF-2α gene [23]. A Renville reporter plasmid (pRL-null) was used as transfection control. Plasmids were transfected into cells using the Lipofectamine and Plus Reagent method (Life Technologies, NY). Cells were pre-treated with rapamycin (20 nM) or AICAR (20 mM) or rapamycin+AICAR. Forty-eight hours after transfection, cells were harvested and luciferase activity was measured using Dual-Luciferase Reporter assay kit (Promega, Madison, WI) and normalized to Renilla activity.

### Electrophoretic mobility shift assays (EMSAs)

Nuclear proteins were prepared from AML cells using nuclear and cytoplasmic extraction kits (Pierce, IL). The protein concentration was determined using the Bradford reagent [22]. EMSA binding reactions were performed as previously described [24]. The oligonucleotides (Akt promoter from −110 to −61 containing Hif2α binding site) were used as forward primer: 5′-CCCCCAGGCACGTGCAGTGGGTCT-3′ and reverse primer: 5′- AGACCCACTGCACGTGCCTGGGGG-3′. The double stranded oligonucleotiudes were end-labeled, and twenty fmol of labeled probe was incubated with the nuclear extracts and 1 μg of poly (dI-dC)·(dI-dC). To test the specificity of HIF1/2α binding to Akt promoter, 5 μl of HIF-1α or HIF-2α antibody (Cell Signaling Technology, Danvers, MA) was pre-incubated with nuclear extracts. The binding reaction was carried out at room temperature for 15 min prior to adding the radiolabeled probe. Competition binding was performed in the presence of a 100-fold excess of the unlabeled oligonucleotide. The complexes were resolved using a 5% non-denaturing polyacrylamide gel. The gels were dried and autoradigraphed.

### Migration and invasion assays

Migration and invasion assay was performed using 24-well dish with the inserts (Millipore, MS). AML cells were seeded into upper chamber and treated with rapamycin (20 nM) or AICAR (2 mM) or drug combinations (20 nM rap/2 mM AICAR) for 72 hrs. The lower chamber was filled with DMEM medium. Following 72 hrs of incubation, each well was washed with PBS and the cells were stained with 0.1% crystal violet solution for 15 min. Following staining; the cells were thoroughly washed in water and air-dried. The total number of migrated and invaded cells was counted using counting software and the images of migrated or invaded cells were taken using Nikon light inverted microscope.

### Animals

#### TSC2^+/−^ mice

TSC2^+/−^ mice were generously provided by Dr. David J Kwiatkowski (Harvard Medical School, MA). The animals were allowed food and water ad libitum prior to and during the experiments. Mice at 12 months old mice were divided into four groups, each group containing four mice. Group 1 (control), mice were injected with an equal amount of DMSO. Group 2, mice were injected with 2-mg/kg-body weight rapamycin in DMSO 5 days/week for 4 weeks [17]. Group 3, mice were injected with 250mg/kg body weight AICAR in DMSO 5 days/week for 4 weeks as previously described [25]. Group 4, mice were injected with same doses of rapamycin (2 mg/kg) and AICAR (250 mg/kg) in DMSO 5 days/week for 4 weeks. The drugs were injected i.p. under isofluorane inhalation anesthesia (Abbott, Abbott Park, IL). Animals were euthanized and kidneys were removed rapidly to measure tumor size by two independent observers blinded to the experimental conditions. The kidneys were removed rapidly for dissection and bio-chemical analysis. Half of kidney from each mouse was formalin fixed, paraffin embedded and tissue blocks were serially sectioned.

### Toxicity assay

Urinary excretion of glutathione-*S*-transferase (GST) an indicator of the loss of cell membrane integrity was used as a marker of kidney toxicity. GST was measured in urine from all mice groups. In addition, GST was also measured in serum from each mouse. GST was measured using dinitrochlorobenzene and glutathione (GSH) as substrate and co-substrate respectively [26]. Final data represents as one unit of GST activity is the formation of 1 μmol of dinitrochlorobenzene GSH conjugate/ml/min at pH 6.5 and 25°C.

### Immunoperoxidase staining of Ki67

Detection of Ki67 was performed on paraffin kidney tumor sections by immunoperoxidase staining [27]. Kidney sections were incubated with rabbit anti-Ki67 antibody (Abcam, Cambridge, MA) for 30 min. After washing in PBS, sections were incubated with horseradish peroxidase labeled anti-rabbit antibody for 30 min. The horseradish peroxidase was developed with diaminobenzidine tetrahydrochloride and hydrogen peroxide in PBS. To demonstrate staining specificity, control kidney sections were stained without primary antibody. Kidney sections were viewed and photographed using a Nikon Research microscope equipped for epifluorescence.

### TUNEL assay

Apoptosis in tissue sections was examined using the TUNEL Apoptosis Detection Kit (Upstate) as previously described [20]. Sections incubated in PBS served as the negative control. The number of TUNEL-positive cells was counted in five randomly selected fields under 40X magnification in each kidney section from each animal.

### Statistics

Data are presented as mean ± standard error. Statistical differences were determined using ANOVA followed by Student Dunnett's (Exp. vs. Control) test using 1 trial analysis. *P-*values less than 0.01 were considered statistically significant.
